# Macronuclear genome structure of the ciliate *Nyctotherus ovalis*: Single-gene chromosomes and tiny introns

**DOI:** 10.1186/1471-2164-9-587

**Published:** 2008-12-05

**Authors:** Guénola Ricard, Rob M de Graaf, Bas E Dutilh, I Duarte, Theo A van Alen, Angela HAM van Hoek, Brigitte Boxma, Georg WM van der Staay, Seung Yeo Moon-van der Staay, Wei-Jen Chang, Laura F Landweber, Johannes HP Hackstein, Martijn A Huynen

**Affiliations:** 1 Centre for Molecular and Biomolecular Informatics, Nijmegen Centre for Molecular Life Sciences, Radboud University Nijmegen Medical Centre, Geert Grooteplein 28, 6525 GA, Nijmegen, the Netherlands; 2 Department of Evolutionary Microbiology, Radboud University Nijmegen, Heyendaalseweg 135, 6525 AJ Nijmegen, the Netherlands; 3 Department of Ecology and Evolutionary Biology, Princeton University, Princeton, NJ 08544, USA; 4 Laboratoire de Biometrie et Biologie Evolutive, University de Lyon University de Lyon 1, CNRS, 43 boulevard du 11 novembre 1918, Villeurbanne F-69622, France

## Abstract

**Background:**

*Nyctotherus ovalis *is a single-celled eukaryote that has hydrogen-producing mitochondria and lives in the hindgut of cockroaches. Like all members of the ciliate taxon, it has two types of nuclei, a micronucleus and a macronucleus. *N. ovalis *generates its macronuclear chromosomes by forming polytene chromosomes that subsequently develop into macronuclear chromosomes by DNA elimination and rearrangement.

**Results:**

We examined the structure of these gene-sized macronuclear chromosomes in *N. ovalis*. We determined the telomeres, subtelomeric regions, UTRs, coding regions and introns by sequencing a large set of macronuclear DNA sequences (4,242) and cDNAs (5,484) and comparing them with each other. The telomeres consist of repeats CCC(AAAACCCC)n, similar to those in spirotrichous ciliates such as *Euplotes*, *Sterkiella *(*Oxytricha*) and *Stylonychia*. Per sequenced chromosome we found evidence for either a single protein-coding gene, a single tRNA, or the complete ribosomal RNAs cluster. Hence the chromosomes appear to encode single transcripts. In the short subtelomeric regions we identified a few overrepresented motifs that could be involved in gene regulation, but there is no consensus polyadenylation site. The introns are short (21–29 nucleotides), and a significant fraction (1/3) of the tiny introns is conserved in the distantly related ciliate *Paramecium tetraurelia*. As has been observed in *P. tetraurelia*, the *N. ovalis *introns tend to contain in-frame stop codons or have a length that is not dividable by three. This pattern causes premature termination of mRNA translation in the event of intron retention, and potentially degradation of unspliced mRNAs by the nonsense-mediated mRNA decay pathway.

**Conclusion:**

The combination of short leaders, tiny introns and single genes leads to very minimal macronuclear chromosomes. The smallest we identified contained only 150 nucleotides.

## Background

Ciliates form an extremely diverse taxonomic group of protozoa. They are the most complex single-celled eukaryotes, with some genomes containing more than 20,000 genes [1], Examples are the two completely sequenced ciliates *Paramecium *(39,642 genes) [2] and *Tetrahymena *(27,424 genes) [3]. They are abundant in almost every aqueous environment, from ocean waters to small ponds and even pockets of soil water; and they can grow as symbionts, commensals and parasites in pelagic, benthic, sapropelic or intestinal ecosystems. However, despite this diversity, ciliates have one common and unique feature: they possess two types of nuclei, each with its own specific function.

The micronucleus of ciliates represents the germ-line and contains high molecular weight DNA in the form of large chromosomes, while the macronucleus encodes proteins and RNAs required for somatic functions [4]. The macronucleus contains DNA on sub-chromosomal fragments, which in *Paramecium *and *Tetrahymena *range from 50 kb to more than 1,500 kb [1,5]. Macronuclei are generated from the post conjugation micronuclei in a complex process that involves DNA elimination and large-scale genomic rearrangements [5-10]. This process and the genetic organization of the macronucleus have been intensively studied in both peritrichous and spirotrichous (formerly hypotrichous) ciliates [5,7,8,11]. In peritrichous ciliates, such as *Paramecium *and *Tetrahymena*, about 20% of the micronuclear DNA is deleted during macronucleus formation, the chromosomes become fragmented and reorganized in sub-chromosomal molecules. In spirotrichous ciliates such as *Stylonychia *or *Sterkiella (Oxytricha)*, the formation of macronuclei is especially complex since it can involve the loss of more than 95% of the complexity of the DNA and large-scale reordering of the protein-encoding DNA segments. Protein-encoding sequences are joined and assembled into the proper open reading frame, a process that depends on maternal guide RNAs [12]. Lastly, the open reading frames (ORFs), together with non-coding 5' – leader and 3' – trailer sequences, are released as individual, short DNA molecules. These molecules are capped with telomeres, and selectively amplified as individual "mini-chromosomes" [8,13,14].

In certain spirotrichous ciliates, the development of the new macronucleus involves transient formation of polytene chromosomes [6,10]. These giant chromosomes resemble the polytene chromosomes of dipteran larvae but are transcriptionally inactive and disintegrate shortly after reaching their highest degree of polyteny [6,10]. Genome rearrangements, DNA elimination and, lastly, reorganization of the residual DNA into "gene-sized pieces" occur during the breakdown of polytene chromosomes.

Here we examine the structure of the macronuclear DNA of *Nyctotherus ovalis*, a member of a third group of ciliates known as the heterotrichous ciliates. *N. ovalis *lives in an anaerobic intestinal environment. It is of particular interest because it possesses hydrogenosomes that contain a genome [15] and that therewith have provided evidence that these organelles evolved from a mitochondrial ancestor [16]. There are some reports [17-20] that the heterotrichous ciliates *Nyctotherus cordiformis *and *N. ovalis*, which live in the intestinal tracts of frogs and cockroaches, respectively, form polytene chromosomes after conjugation, similar to *Stylonychia *[21]. With respect to the structure of the *N. ovalis *macronuclear chromosomes there is evidence from studies on individual genes, such as those for hydrogenase [10] and alpha tubulin [22] from telomere-capped sequences [16], as well as from a set of 34 complete macronuclear chromosomes [23], that *N. ovalis*, like *Stylonychia*, has gene-sized chromosomes. Alignment of the macronuclear chromosomes with known proteins furthermore has indicated the presence of short (< 30 nt) introns [15,16,22,23]. We examined the structure of *N. ovalis *gene-sized chromosomes and the regulation of their transcription by combining information from large-scale cDNA (4,242 sequences) and cDNA libraries (5,484 sequences, including 327 complete macronuclear chromosomes). *N. ovalis *possesses very minimal macronuclear chromosomes, with short leaders, tiny introns and single genes. The introns tend either to contain in-frame stop codons, or their length is not dividable by three. *N. ovalis *thus prevents translation of unspliced mRNAs, as has been described for *P. tetraurelia *[24].

## Results

### 1.1 Chromosome structure

#### 1.1.1 Gene-sized chromosomes

**Figure 1:**
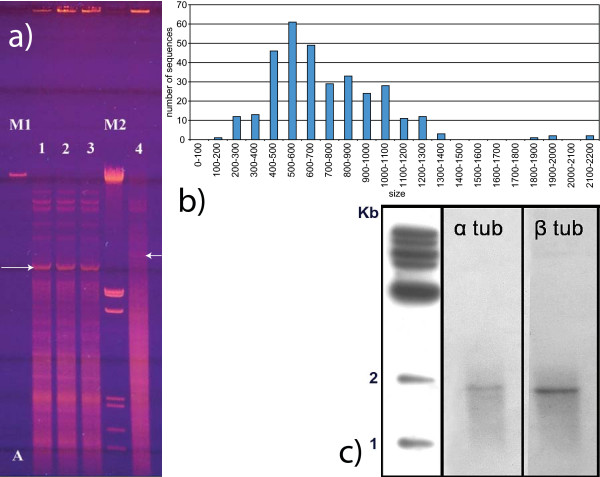
**a) Structure of *N. ovalis *macronuclear chromosomes. 1a-0.4% Agarose gel: M1 = λ- DNA (48502 bp).** M2 = λ Eco RI/HindIII (sizes of the DNA fragments: 21226, 5148+4973, 4268, 3530, 2027, 1904, 1584 and 1375 bp). Lane 1,2 and 3 contain total DNA of *Nyctotherus ovalis*. Lane 4 contains total DNA of *Euplotes crassus*. Arrows indicate the ribosomal clusters (see Materials). b) Length distribution of the sequenced full-length macronuclear chromosomes. c) Southern blotting: unrestricted total DNA of *N. ovalis *was separated on a 0.7% agarose gel, blotted and hybridized with clones containing the α2 tubulin (α tub) and the β2 tubulin gene of *Stylonychia lemnae*. The labeling of single, gene-sized bands indicates the presence of tubulin genes on gene-sized DNA molecules.

We surveyed the genomic organization of the macronucleus of *N. ovalis*. Agarose gel electrophoresis revealed that the majority of the undigested DNA isolated from intact *N. ovalis *was present in the form of low molecular weight DNA (size range 0,5–10 kb; Figure 1; [15]). Southern blotting revealed that genes were present as gene-sized DNA fragments (Fig. 1, [10]). To survey the genome organization of *N. ovalis *at the sequence level, we generated a macronuclear gDNA library and sequenced 4,242 representative clones, as well as 5,484 cDNAs from a polyA-plus cDNA library. It should thereby be noted that *N. ovalis *cannot be cultured yet. Cells have to be isolated by hand from dissected hindguts of cockroaches. Only a small number of cockroaches contain *N. ovalis *cells at a density of several hundred cells per hindgut, making it very difficult to obtain sufficient material for DNA/RNA isolation. Therefore, performing a total sequencing of the genome and the corresponding cDNAs will therefore be extremely difficult if not impossible. In the current study we obtained 4,755 non-redundant gDNAs and cDNAs with a limited overlap between them. Assuming a genome size similar to the published Ciliate genomes (27,000 and 40,000 genes) this corresponds to ~15% of the total genome. Randomly selected clones were sequenced in a single reaction. After filtering vector sequences and poorly-resolved terminal regions (see Materials section), we clustered the remaining sequences to remove redundancy. Grouping sequences with ≥ 97% identity over a stretch of at least 100 nucleotides resulted in our identifying 2,841 gDNA clusters (692 containing between two and 23 sequences and 2,149 single sequence clusters) and 1,914 cDNA clusters (416 containing between two and 843 sequences and 1,498 single sequence clusters). These will be referred to as the nr-set (non-redundant set). Sequences with non-determined nucleotides (N) were excluded from analyses that required correct nucleotides at all positions, like genetic code analysis (see below).

In the 2,841 gDNA nr-set we identified 327 sequences that were capped by telomeres at both ends, representing complete macronuclear chromosomes. In none of these could we find evidence for the presence of more than a single gene (see Materials). Moreover, we examined carefully all the incomplete gDNA sequences for multiple genes containing macronuclear chromosomes. Here too we only observed a single gene per chromosome. The vast majority of the macronuclear chromosomes were smaller than 7 kb, and the sequencing of clones with inserts less than 6–7 kb did not provide any evidence for the presence of more than one gene per chromosome. However, there were DNA bands (Figure 1a) larger than 10 kb that were neither cloned nor sequenced. Therefore, we cannot exclude the possibility that a few chromosomes with several genes were present. Similarly to the protein-coding chromosomes, the nr-set was found to contain 12 separate chromosomes coding for single tRNA molecules. We will refer to this pattern as a "one macronuclear chromosome one-transcript rule" rather than a "one-macronuclear chromosome one-gene rule", because the complete ribosomal array (28S, 5.8S and 18S rRNA genes) is located on a single macronuclear chromosome and the complete ribosomal RNA tends to be encoded on a single transcript in ciliates [8].

We examined the macronuclear chromosome length distribution for all 327 fully-sequenced chromosomes (Figure 1b). The smallest two-telomere-capped sequence we found that encodes a known gene was a serine tRNA (AM890335), with 233 nucleotides between the two telomeres. The smallest two-telomere-containing sequence we found for which no homologs could be identified was 150 nt (accession number AM890150). This sequence is the smallest putative macronuclear chromosome ever identified (although, one should keep in mind that these molecules lack centromeres, unlike true chromosomes). The presence of this 150 nt macronuclear chromosome was confirmed by nested PCR with specific primers. It does not contain a substantial open reading frame and might therefore code for a structural RNA. However, analysis of its RNA secondary structure did not indicate a significantly lower free energy than for random sequences with the same base composition, nor did analysis with miRNA prediction programs indicate a miRNA like structure (see Materials).

#### 1.1.2 Telomere sequences

We sequenced 13 full length macronuclear chromosomes using the same approach applied to other species [25]. Telomeres were observed with the sequence CCC(AAAACCCC)_*n *_at the 5' end, and the sequence (GGGGTTTT)_*m*_GGG (plus one of the form (GGGGTTTT)_*m*_GGGGTTT) at the 3' end, with *n *and *m *ranging from 1 to 6. There appeared to be a trend of one long and one short telomere, giving rise to a negative Pearson product-moment correlation coefficient between the number of repeats in 5' and 3' telomeres (-0.83925). This contrasts with the report of Cavalcanti *et al. *[25], who observed no clear trend in the lengths of telomeres in *Sterkiella histriomuscorum (Oxytricha trifallax)*. However, the libraries were generated differently and our initial data set of 13 sequences was small compared to the 1,028 clones in ref [25].

Using the motifs determined above, we identified telomeres on 2,249 of the 2,841 gDNA sequences. Of these sequences, 327 contained telomeres on both sides, suggesting that they represented complete macronuclear chromosomes. Several (larger) chromosomes carrying a single gene have been sequenced manually using suppression PCR as in [26]. Because we used telomere primers for amplification of the total gDNA for the generation of the gDNA library, we could not determine the length of the telomeres in the set of 327 exactly, as primer-shifting occurs during the PCR.

#### 1.1.3 Subtelomeric regions

We define the chromosomal leader as the subtelomeric region that runs from the 5' telomere to the beginning of the ORF and the chromosomal trailer as the subtelomeric region that runs from the stop codon to the 3' telomere. In order to identify these regions, we first oriented 1,459 gDNAs by predicted homology to a known protein in UniProt (SWX, e-value threshold = 1.e-05). Subsequently we compared these gDNAs with the 1,914 nr-cDNAs using GeneDetective (see Materials) followed by manual checking of all cDNA/gDNA pairs. Combining these data with the putatively homologous proteins from UniProt, we could infer the position of 14 ATG start codons precisely. The size of the leaders in these 14 chromosomes ranged from 60 to 184 nucleotides, with an average of 112, while the length of the 5'UTRs ranged from 8 to 44 nucleotides. We followed the same procedure to identify the trailers and 3' UTRs: using the cDNAs with a match against the gDNAs and against a known protein we could accurately identify eight regions running from the stop codon until the polyA addition site. The 3' UTR length ranges from 13 to 68 with an average of 36 nucleotides. TAA was generally used as stop codon. The size of the chromosomal trailers ranged from 33 to 125 nucleotides.

To examine whether there were any biases in the nucleotide composition of the subtelomeric regions, we calculated the Shannon entropy, the purine skew, and GC skew for the 300 first nucleotides after the 5' telomeres and for the last 300 nucleotides before the 3' telomeres.

**Figure 2:**
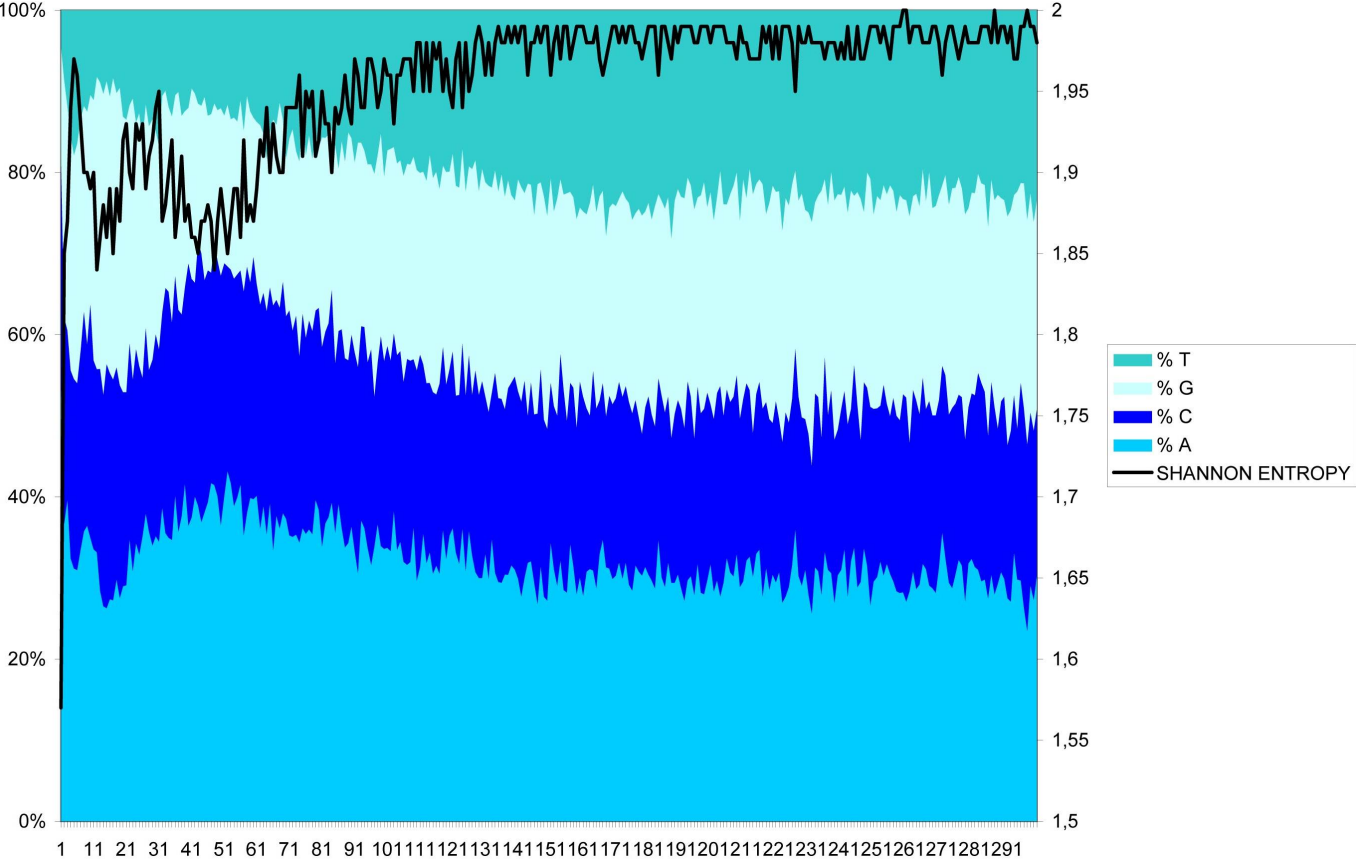
Nucleotide frequency distributions and Shannon entropy of the frequency distribution of the first 300 nt after telomeres in the 5' of the coding strand.

761 sequences were found to contain at least 300 nucleotides after the 5' telomere and to have a BlastX hit in UniProt that allowed determination of the coding strand. The Shannon entropy and the frequency of each base are plotted in Figure 2. For four different states (in our case, four nucleotides), the maximum entropy is two, corresponding to equal nucleotide frequencies. Lower entropies indicate a bias in the composition.

**Figure 3:**
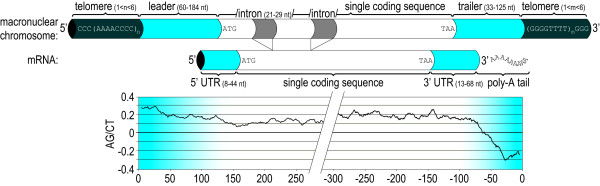
**Schematic view of *N. ovalis *macronuclear chromosome structure and the purine/pyrimidine ratios.** Lengths of the various parts composing the chromosome and cDNA are displayed. The combination of short leaders, short introns and single genes leads to very short chromosomes. In this figure we can observe as well that the purine/pyrimidine skew is higher than 0 in the coding strand, allowing the prediction of coding strands for chromosomes without a homolog among known proteins.

The results indicated a bias in the nucleotide frequencies in the first 70 bases that was found to disappear at about 130 nucleotides. The region of the bias coincided with the length of the 5' leaders (between 60 and 184 with an average of 112). To explain the low entropy, we examined different types of biases in the nucleotide composition. We observed the strongest bias in the purine/pyrimidine ratio, expressed in the so-called purine skew ((A+G)-(C+T))/((A+G)+(C+T)). This index varied from -1 (only C and/or T) to 1 (only A and/or G), with a value of 0 corresponding to no skew. More purines than pyrimidines were found in the 5' region of the coding strand (Figure 3), especially in the first ~130 nt.

There were 505 sequences containing at least 300 nucleotides before the 3' telomere, having a BlastX hit in UniProt that allowed determination of the coding strand. As with the 5' ends of the chromosomes, the Shannon entropy indicated a bias in the last ~70 nucleotides, but in contrast to the 5' leader, a low percentage of G and a general pyrimidine skew. In other words, symmetry was found in the purine skew. Whether one starts from the 5' end of the coding strand, or from the 5' end of the non-coding strand, there is a strong purine skew near the telomere. The pyrimidine skew at the 3' end of the chromosomes did not, however, extend into the coding region, which showed a general purine skew (Figure 3). This allowed us to predict which strand was coding in chromosomes without detectable homology to a known protein (see below). The purine skew in the subtelomeric regions has also been described for the ciliate *Sterkiella *[25]. The skew represents a violation of parity rule two, which states that complementary DNA strands tend to have the same purine/pyrimidine composition [27-29].

#### 1.1.4 Intron structure

**Figure 4:**
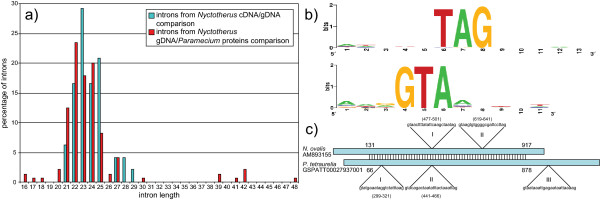
**a) Distribution of the intron length (in percentage) within the two sets of introns: The experimental set (48 introns) that was obtained by comparing *N. ovalis *cDNAs with gDNAs and the predicted set (145 introns) that was obtained by comparison of *N. ovalis *gDNAs with *P. tetraurelia *proteins.** The predicted set contains three longer introns that are not shown in this histogram (76, 114 and 252 nt long). b) Schematic representation of the exon/intron boundaries with WebLogo in 48 introns. The GTA and TAG motifs are well conserved. c) Conservation of intron location between *N. ovalis *and *P. tetraurelia *in two genes that are orthologous to Succinyl-CoA ligase. Introns were considered conserved when they were within 2 amino acids of each other in the protein alignment. Intron I of *N. ovalis *and II of *P. tetraurelia *are conserved. Intron II of *N. ovalis *and I of *P. tetraurelia *are not conserved. Whether Intron III of *P. tetraurelia *is conserved or not cannot be determined.

To detect introns, we first performed a comparison between the cDNA and gDNA clustered sets using GeneDetective (Materials). After removing paralogs and refining the intron boundaries by hand, we identified a total of 48 introns in 27 gDNAs. This intron set will be referred to as the experimentally-derived introns. All introns were very short, ranging from 21 to 29 nucleotides with an average of 24 nt and a median of 23 nt (Figure 4a). Intron prediction based on the comparison with *P. tetraurelia *proteins (see below) gave an identical median of 23 nt but a larger average of 26 nt, due to a tail of a few relatively long introns (Figure 4a). This is similar to the average intron length of 27 nt (ranging from 21 – 66) reported by McGrath et al.[23], which was based on comparison of gDNAs with known proteins. Comparison of the average intron length (27) with the intron length range (21–66) in the analysis of McGrath et al., indicates their intron length distribution also has a long tail. The existence of these "long" introns (> 30 nt) is weakly supported, as we do not observe them in our more direct gDNA/cDNA based comparisons. Furthermore, by doing BlastX comparisons with a larger database (UniProt) we could correct 8 of the 15 long (11 introns ≥ 30 nt) and very short introns (4 introns ≤ 18 nt) that were predicted by the comparison with *P. tetraurelia *to fall within the boundaries that were identified for the experimental introns (21 to 30 nt). This underscores that intron prediction by comparison against a protein database depends critically on the proteins in that database.

The boundaries of the experimentally derived introns are very similar to each other: exon|GTA......TAG|exon; where "|" is the border between exon and intron (Figure 4b), with only two exceptions for which the last three intron nucleotides are CAG and TGG. Examination of the introns with AlignACE [30], a program that finds conserved motifs, did not identify a branch point or any other overrepresented motifs other than the intron boundaries. The introns contain a high percentage of thymidine (35%) in the introns versus 23% in the coding sequence. Guanine (17% vs. 24%) and cytidine (17% vs. 21%) are low, while adenine (31% vs. 32%) is more or less the same in the coding regions and the introns. This is different from what we observe for other non-coding sequences such as the leaders, where the thymidine content was lower than in the coding sequence. The introns in protein-coding sequences appear to be of the type that is excised by the spliceosome. Supporting the presence of a standard mRNA splicing machinery, we identified seven homologs of members of the spliceosome complex: Splicing factor 3B subunit 1, Splicing factor 3A subunit 3, splicing factor YT521, Splicing factor 1, splicing factor SLU7, Spliceosomal U5 snRNP-specific 15 kDa protein and Splicing factor U2AF 35 kDa subunit.

#### 1.1.5 Intron conservation

To examine the evolutionary conservation of the intron positions, we compared the 48 introns of the 27 *N. ovalis *gDNA sequences (above) with those of their closest homologs in the ciliate *Paramecium tetraurelia*. The latter also has tiny introns (comprising between 20 and 33 nucleotides [31]) for which a large number have been experimentally determined (Materials). Of the ten introns in *N. ovalis *sequences with significant best hits in *P. tetraurelia*, in which the aligned regions "covered" the *N. ovalis *introns, four occur at the same position in *P. tetraurelia*. A typical example is the succinyl CoA ligase gene that contains two introns in *N. ovalis*. One of these is conserved in *P. tetraurelia*, which itself has two introns in the region that can be confidently aligned between the two species (Figure 4c). To our knowledge this is the first observation of the conservation of these tiny introns. In order to quantify the level of intron conservation, we performed a second analysis in which we compared the *N. ovalis *gDNAs in which intron locations were predicted using Genewise [32] (Materials) with proteins with experimentally determined (gDNA/cDNA) intron locations in *Paramecium tetraurelia *(these were kindly provided by Laurent Duret [33]). Here we observed conservation of the intron position for 48 introns between *P. tetraurelia *and *N. ovalis*, out of 145 introns that Genewise predicted in *N. ovalis *based on comparisons of the *N. ovalis *gDNAs with the *P. tetraurelia *proteins. The fraction of conservation of tiny introns between these ciliates therefore appears to be about 1/3.

Jaillon et al. [24] have reported that introns which lack in-frame stop codons tend not to occur in lengths dividable by three. This guarantees that unspliced mRNAs will be recognized by the NMD by having premature stop codons, either in the intron itself or caused by the frame shift resulting from the retained intron. Such a trend is also visible in the *N. ovalis *predicted introns. Out of the 145 introns, 40 do not have an in-frame stop codon (TAA, TAG or TGA, see below). Out of these 40 only 3 have a length that is dividable by three. In contrast, of the 105 introns that do have an in-frame stop codon, 60 have a length dividable by three. There is thus, among the introns without in-frame stop codons, a significant under-representation of introns dividable by three compared to introns that do contain stop codons (P = 1E-7, based on the chi squared test).

The conservation of tiny introns between the very distantly-related *P. tetraurelia *and *N. ovalis *indicates that some introns can be traced back to early ciliate evolution. We also examined conservation of intron positions with *Tetrahymena thermophila*, which does not contain tiny introns (containing between 53 and 978 nt, see Figure 6). In the sequence alignments, the positions of three *T. thermophila *introns lay within 2 amino acids of introns in *N. ovalis *[see Additional file 1]. This would argue for independent evolution of intron position and intron length. Alternatively, in ciliates with tiny introns it is possible that intron length is strongly reduced during the development of the macronucleus. In any case, the total number of conserved introns for which there is experimental evidence in at least one species is small, and precludes definitive statements regarding their independent evolution from genome structure. We could not detect any conservation of intron positions in the more distantly-related alveolate *Plasmodium falciparum*. We searched for gDNAs resulting from bacterial to ciliate or archaeal to ciliate Horizontal Gene Transfer events using a similar method as we used for rumen ciliates [34]. In this set we found no evidence of introns.

With a length between 21 and 29 nucleotides and an average of ~24 nucleotides, the introns in the *N. ovalis *set described in this report are among the smallest spliceosomal introns known. The shortest spliceosomal introns, 18 nucleotides, have been described in the nucleomorph of a chlorarachniophyte algae [35]. Several short introns have been described in ciliates in the literature. For example, the introns in *P. tetraurelia *are between 20 and 33 nt [31], and the introns in *Sterkiella histriomuscorum *are between 31 and 137 nt in length [36]. In *Euplotes octocarinatus*, introns were detected in a range of 30 to 145 nt [37,38]. Four short introns, ranging from 37 to 47 nt, were also described in gamma tubulin encoding genes of *Euplotes crassus *[39].

### 1.2 Transcriptional properties

#### 1.2.1 Orientation of the sequences

The macronuclear chromosomes we sequenced contain just one gene. Being able to determine the coding strand of these sequences would be a first step in annotating those sequences without hits against known proteins. We used the observation that the coding strand has a positive purine/pyrimidine ratio (Figure 3) to orient the sequences that lack hits to known proteins. Two other methods that we applied were: 1) finding the best ORF using getORF from EMBOSS and 2) calculating the GC gradient [40] on the set of 1,459 sequences having a match against a known protein from UniProt (UniProt set), as a benchmark. The purine/pyrimidine ratio was the best indicator of the coding strand with a good prediction in 97% of the cases, compared to 80% for the ORF prediction and ~50% for the GC gradient. Using the purine/pyrimidine ratio allowed us to orient all 1,382 sequences that did not belong to the UniProt-set with 97% confidence.

#### 1.2.2 Motif discovery

In all ciliate species with gene-sized macronuclear chromosomes which have been studied, approximately 95% of germ-line DNA is eliminated during the development of the macronucleus [41]. The incredibly short leader and trailer that remain must therefore contain a high density of signals for processes like telomere addition, replication, copy regulation and transcription. Thus, ciliates provide a uniquely compact model to study these basic eukaryotic regulatory systems. Because we identified homologs of a number of transcription factors (E2F5 family transcription factor, transcription factor Dp-2, MADS box transcription factor, C-MYB-like transcription factor and transcription factor E2F/dimerisation partner (TDP)) in our data set, we can also expect their regulatory elements to be present in the *N. ovalis *macronuclear genome. We searched our nr-set of *N. ovalis *sequences for novel motifs and known Transcription Factor Binding Sites (TFBS).

As genes with related functions are often co-regulated, they may be expected to contain comparable signals in their non-coding DNA [30,42]. Therefore, to search for meaningful motifs, we first composed groups of related genes based on a number of different criteria. Genes were clustered based on membership of an orthologous group (OG, see Materials) to detect signals shared by paralogous genes, or on the membership of a common pathway. For the latter we used both the KEGG pathways and EcoCyc pathways. In addition we composed a set of 12 non-redundant tRNAs identified with tRNAscan-SE [43]. For each group of sequences (except for tRNAs, see below), we searched the first 200 nucleotides after the 5'-telomere using AlignACE [30] and MEME [44]. Note that 200 nt should be enough to cover the complete upstream non-coding DNA, even in the sequences where no translation start site could be identified (Figure 3).

Using this approach, we could identify several potential regulatory motifs. First, MEME identified a motif with the consensus sequence AATTAACATGAGTC in the upstream regions of 12 Chaperonin GroEL genes of the HSP60 family (COG0459; the data set contained the alpha, beta, eta, theta, gamma and epsilon subunits and HSP60). Searching this motif with MAST, we found that it also occurs upstream of a protein from the superfamily DNA and RNA helicase, with the motif profile fitting the helicase sequence with an e-value of 3.6e-06. HSP60 and helicase could be regulated concomitantly as they can both be regulated by DNA-dependent protein kinase [45,46]. Another study also reported that dysregulated expression of an RNA helicase leads to an altered distribution of HSP60 [47].

**Figure 5:**
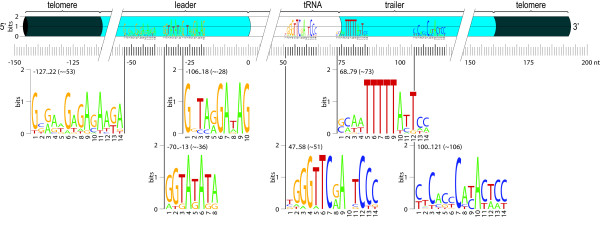
**Schematic view of the structure of tRNA macronuclear chromosomes.** The motifs found with MEME in more than half of the 12 nr-tRNAs are displayed as a WebLogo picture, together with their positions (from..to (~average)). 0 corresponds to start of the tRNA.

Second, we identified conserved motifs in the tRNA chromosomes, by searching the entire set of gene-sized tRNA chromosomes (Figure 5). Two of the motifs we identified have very well-conserved positions: a likely TATA box (~35 nt upstream of the tRNA), and a motif with consensus GCAATTTTTATTCC (~2 nt downstream). This last motif corresponds to the termination signal of RNA polymerase III transcription [48,49]. Searching for these motifs in the whole nr-set using MAST [50] identified no other sequences that contained these motifs, which is not surprising, as the protein coding genes are transcribed by RNA polymerase II. The TATA box could be identified in some sequences that we checked manually.

In an additional search for known TFBS, we used MAST [50] to search for the 398 Transfac MATRIX 7.0 motifs [51]. These results did not link sets of functionally related sequences (e.g. shared KEGG pathway or COG functional category, not shown).

Considering the few motifs identified in these analyses, it should also be noted that the Transfac profiles we searched for are specific for several eukaryotic species but not for ciliates. Furthermore, previous studies of spirotrichous ciliates have identified few conserved motifs, leading to the suggestion that telomeres may also play a role in regulating gene expression/DNA replication [52].

In the trailer region we searched for the consensus eukaryotic polyadenylation signal AATAAA [53,54]. This motif was not found in our sequences, confirming a previous study on a smaller dataset [55]. Examining by hand the eight pieces of trailer sequences identified with the help of cDNAs and known homologs [see Additional file 2] revealed no degenerate motif either. McGrath and co-workers [23] have reported an over-represented long motif in the 5' and 3' ends in their set of 34 complete chromosomes, and a shorter subsequence of that motif in the 5' and 3' ends of all the chromosomes. Neither the consensus sequence of the long motif, nor its shorter subsequence was present in our dataset, nor could we detect significant similarity to the consensus sequences in our gDNAs.

#### 1.2.3 Genetic code

In a set of 328 cDNAs that could confidently be aligned with the C-terminus of known proteins, TAA was most often the first codon after the C-terminus of the protein alignment (141 cases), and therewith obviously the stop codon. Consistent with earlier findings that were based on smaller sets of sequences [23,55] we conclude that *N. ovalis *preferentially uses UAA as a stop codon. UAG was rarely observed in the stop codon position (in 5 out of 328 cDNAs) and UGA was never observed in the stop codon position. When reexamining the published sequences from McGrath et al.[23] and Destables et al.[55] we noticed that when using this strict criterion of requiring an alignment until the end of the C-terminus of a known protein and only considering as potential stop codon the first codon 3' of the protein alignment, also in these sets TAA was the only stop codon that could confidently be assigned. Nevertheless, UGA and UAG are, like UAA, avoided in cDNAs that could be aligned with known proteins or protein domains. Ciliates have a wide variety of genetic codes [56,57]. For example, several deeply-diverging lineages, including the genera *Tetrahymena *and *Paramecium, Stylonychia *and *Sterkiella*, and *Diophrys *[58] may have independently reassigned UAA and UAG to glutamine, using UGA as their only stop codon [59]. On the other hand, *Euplotes *translates UGA into cysteine and uses UAA and UAG as stop codons[60], while UGA encodes tryptophan in the independent lineages including *Blepharisma *and *Colpoda *[61]. *Nyctotherus *thus seems most similar to Euplotes, using predominantly UAA, and sometimes UAG as a stop codon. The absence of strong positive evidence for the usage of UAG and UGA is consistent with a previous claim [57] that *Nyctotherus *might represent an evolutionary intermediate between the full standard genetic code and a variant code. We could, however, find no evidence for the usage of UGA or UAG as a sense codon, and combined with earlier findings of the usage of UAA and UGA as stop codon [23] this argues for a standard genetic code in *N. ovalis*.

## Discussion and conclusions

### Evolution of macronuclear gene structure

**Figure 6:**
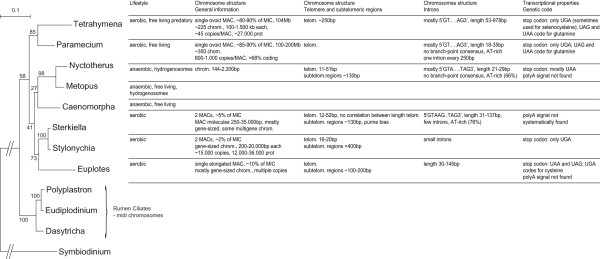
**Phylogenetic tree of 18S RNA from the small ribosomal subunit of the most-studied ciliates with respect to their macronuclear structure.** The tree was generated with PhyML with 100 bootstraps. The dinoflagellate *Symbiodinium *is used as an outgroup to root the tree. *N. ovalis *has a comparable macronuclear structure to *Spirotrichea*, its sister group in the tree (the lineage containing *Euplotes *through *Sterkiella*). MAC stands for macronucleus, MIC for micronucleus.

To analyze the evolution of ciliate macronuclear genome structure we performed a phylogenetic analysis of the 18S RNA from the small ribosomal subunit from various ciliates (Figure 6). The *Clevelandelids *(*Nyctotherus*) form a monophyletic group with the *Armophorea *(*Metopus*, *Caenomorphidae *gen. sp.) and are a sister group of the *Spirotrichea *that contains *Sterkiella (Oxytricha)*, S*tylonychia *and *Euplotes*. Despite their differences in ecology (respectively anaerobic and aerobic), these two sister groups share many features, as already mentioned [3,6,8,15,16,21,25,31,52,62-67]. Another class of ciliates, namely the *Phyllopharyngea*, also possess gene-sized macronuclear chromosomes, but they are not monophyletic with the *Spirotrich*/*Clevelandelids*/*Armophorids *[22]. 'Invention' of gene-sized-macronuclear chromosomes has therefore either preceded their taxonomic separation or more likely arisen several times in evolution. Rumen ciliates that are included in the cluster of 'fragmented' macronuclear chromosomes have much larger (40–80 kb) macronuclear chromosomes with many genes per chromosome (Hackstein, unpublished data). They may represent an intermediate between the '*Tetrahymena*/*Paramecium*" type of long macronuclear chromosomes and gene-sized-chromosomes. The presence of tiny introns does not correlate with the presence of mini-chromosomes. Rather, the exact conservation of the location of a substantial number of the tiny introns between *N. ovalis *and *P. tetraurelia *argues for independent evolution of the length of macronuclear chromosomes and intron size. We currently have scarce information about the germ-line micronuclear genome of *N. ovalis*. However, all the items discussed above reveal a striking similarity with the macronuclear genome organization of the spirotrichous ciliates [8,13,59,65]. Codon usage suggests that *Nyctotherus *may be more closely related to *Euplotes *than to the *Oxytrichids*; however there is no evidence that it has a non-standard genetic code.

## Methods

### Hybridization

The α2-tubulin clone [68] and the β2-tubulin clone [69] from *Stylonychia lemnae *were labeled with α-[^32^P]-dATP by PCR with M13-forward and -reverse primers.

Genomic DNA from *Nyctotherus *was separated on a 0.7% (w/v) native agarose gel and blotted onto a Hybond N+ membrane (Amersham) by downward alkaline southern blotting. The filter was pre-hybridized for 1 h at 65°C in hybridization solution (6× SSC, 5× Denhardts solution, and 0.5% SDS (w/v)). After pre-hybridization, the hybridization solution was refreshed and the ^32^P-labeled probe was added. The filter was hybridized at 65°C for 16–19 h. The hybridized filter was washed twice in 2× SSC, 0.1% SDS (w/v) and twice in 0.5% SSC, 0.1% SDS (w/v) at 65°C. Depending on the signal on the filter an extra washing step with 0.2% SSC, 0.1% SDS at 65°C was included. Autoradiograms were exposed for 0.5–4 h at -70°C with intensifying screens.

### Construction of libraries and sequencing

gDNA of *Nyctotherus ovalis *was prepared by dissolving living *Nyctotherus ovalis *cells, purified by electromigration [70], in 8 M guanidiniumchloride. After extraction with phenol/chloroform/isoamyl alcohol (25:24:1) a portion was analyzed on a 1% agarose gel and 5 fractions of different "size" were isolated. Every fraction was amplified by PCR with telomere primers (5'-CCAAAACCCCAAAACCCCAA-3'). Fractions with fragments < 2 kb with taq polymerase from Sigma (elongation time 2'30"), fractions > 2 kb with the "expand long template PCR system" from Roche (elongation time 4'00").

The PCR products were purified on Sephacryl-500 (Amersham), extracted with phenol/chloroform and precipitated. These fragments were ligated in PGEM T-easy (Promega) and transformed in competent *E. coli *Electromax DH10B cells. The 1–2 kb and 2–4 kb fractions contained sufficient clones (10,000) and were used for sequencing.

For the reconstruction of the ribosomal RNA cluster, the band (indicated by an arrow in lanes 1, 2 and 3 of Figure 1a) was cut out of gel and the DNA from this band was isolated with a gel extraction kit (QIAEX II DNA Gel Extraction Kit, Qiagen). A part of this DNA was digested with EcoR1 and cloned in a pUC-18 cloning vector. The obtained clones were sequenced and the largest part of the ribosomal cluster was reconstructed. With a set of specific telomere primers in combination with internal primers the complete ribosomal cluster was obtained.

The cDNA library was prepared from polyA-plus RNA of *Nyctotherus ovalis *with the "cDNA library Construction" kit from BD Biosciences/Clontech according prescription. The cDNA was ligated in the vector pDNR-LIB and transformed in *E. coli *Electromax DH10B cells (Invitrogen). Titer: 2.58.10^7^. Randomly picked clones were selected for sequencing.

5,484 ESTs (Expressed Sequence Tags) and 4,242 gDNAs of *N. ovalis *were sequenced. Vectors and linkers were automatically filtered out of sequences. First the vector pieces were detected using a BlastN search against their respective vector sequences on TimeLogic Decypher machine  using the same parameters as VecScreen (-q -5 -G 3 -E 3 -e 700 -Y 1.75e12 -F mD).

Regions showing similarity to the vector were trimmed from the ends of the sequences. If there was a remaining vector in the middle, it was removed and the longer of the two remaining fragments was kept.

### Data storage and clustering

The sequences and the results of the analyses performed were stored in a relational MySQL database. In order to minimize the redundancy of the data, we aligned the dataset to itself using SWN (Smith-Waterman comparison at the nucleotide level). Sequence pairs having an identity ≥ 97% and an overlap of at least 100 nt were considered to be from the same gene and were put in the same cluster. Sequences having less or no sequence similarity can be in the same cluster as long as they are linked via other sequences. gDNAs telomeres were trimmed prior to the SWN comparison. However, information about these telomeres was kept associated to the sequence name in the database.

All sequence comparisons were run on a Decypher TimeLogic using the Smith-Waterman algorithm for pairwise sequence comparisons and Hidden Markov Models for sequence-to-profile comparisons, active motifs included. The output was subsequently analyzed in a Linux environment using Perl scripts.

### Datasets

We started with 9,726 sequences (5,484 cDNAs and 4,242 gDNAs). From these we used specific subsets that were more particularly designed to answer each specific biological question i.e., the best compromise between using as much data as possible and using good quality data.

We used the following datasets:

- **Initial-set**: contains the clones that were initially sequenced: 4,242 gDNAs and 5,484 cDNAs.

- **Nr-set**: contains the longest sequence of each cluster generated from the Initial-set: 2,841 gDNAs and 1,914 cDNAs.

- **UniProt-set**: contains all *Nyctotherus *gDNAs having homologs with the known proteins from UniProt: 1,459 gDNAs

- **UniProt-nr-set**: contains *Nyctotherus *gDNAs from the nr-set having homologs within the known proteins from UniProt: 937 gDNAs.

- **Princeton-set**: contains 13 carefully sequenced full-length *Nyctotherus *macronuclear chromosomes.

All sequences have been submitted to the EMBL nucleotide database. The numbers are: AM894321–AM899804 for the cDNAs, AM890408–AM894321 for the gDNAs and AM890081–AM890407 for the set of completely sequenced macronuclear chromosomes.

### Telomere removal

From the Princeton-set (see above), we could determine that macronuclear telomeres of *N. ovalis *are mostly of the form CCC(AAAACCCC)n or (GGGGTTTT)mGGG with n and m comprised between 1 and 6. However this set only contains 13 molecules and we found longer telomeres within the Initial-set. Despite the occasional presence of more repeats in this set, the form of these telomeres remains the same, so we identified telomeres by the following regular expression in Perl:/^GTN([CAN]CCCC)/and eventually refined their removal by hand.

### Verifying that macronuclear chromosomes contain a single gene

To determine whether or not the *N. ovalis *sequences contain only one gene, we compared the 2,841 gDNA from the nr-set against UniProt release 9.1 database, with a SWX search and an e-value threshold of 1e-05. For all *N. ovalis *gDNAs, we retrieved the boundaries of the "best hit" to a known protein. Subsequently we examined whether, in the hit list there were any hits that did not overlap with the first hit. We never observed cases of such non-overlapping hits, arguing for the presence of only a single gene per macronuclear chromosome.

### Orientation of the sequences

#### UniProt detection

Sequences were compared against UniProt database (SWX; e-value threshold = 1e-05). Sequences with a match on the reverse strand were reversed and in the case of the presence of one or the two telomeres, the upstream and/or downstream regions were extracted. 1,459 gDNAs could be oriented this way representing 937 different clusters/non-redundant sequences.

#### Purine/pyrimidine ratio

As we discovered that the coding strand had a higher purine/pyrimidine ratio than the reverse strand, we used this ratio to orient the sequences that could not be oriented with UniProt hit. Sequences having a purine/pyrimidine ratio < 1 were reversed.

### Analysis of subtelomeric regions

The 300 first nucleotides after the telomeres 5' and the 300 nt before the telomeres 3' from the sequences of the UniProt-set were extracted. We measured the Shannon entropy (H = $\sum_{i=1}^{n}p(i)\log_{2}(\frac{1}{p(i)})$), the purine skew, defined as ((A+G)-(C+T))/((A+G)+(C+T)), the AT skew, defined as ((A+T)-(G+C))/((A+T)+(G+C)), and the single nucleotide composition of these sequences for the 300 aligned positions.

We searched for conserved motifs using a Gibbs sampling program AlignACE [30], and the motif discovery tool MEME [44] in these regions.

### Intron detection

1,914 cDNAs were compared to 2,841 gDNAs of the nr-set using GeneDetective with the default values except "filter off" on Decypher and Exonerate [71], with the following parameters: model = est2genome, percent = 50 and mini-intron = 20 and were then validated by hand. Exonerate only identified introns in 17 sequences, whereas GeneDetective found an additional 10 sequences with introns. To compare nucleotide percentages in the introns with the rest of the sequences, we used for the latter the pieces of cDNAs matching the gDNAs. Intron boundaries were displayed using WebLogo [72]. Introns in *Nyctotherus *gDNAs were predicted with Genewise [32]. First, all *Nyctotherus *gDNAs were compared with *Paramecium *proteins for which experimentally-derived (gDNA/cDNA comparison based) introns were known, using SWX (Smith-Waterman X) on a Decypher machine. Subsequently GeneWise was run for the best hits of *Nyctotherus *sequences with the *Paramecium *proteins in the "-splice flat-intron tied" mode to identify likely introns in the *Nyctotherus *sequences. From the GeneWise results we extracted the locations of the *Nyctotherus *introns in the *Paramecium *proteins. The latter were then compared with the experimental locations of the *Paramecium *introns to establish which ones were conserved. Introns were considered conserved when the *Nyctotherus *introns mapped to the same location in the aligned *Paramecium *proteins as the *Paramecium *introns, allowing for a maximum shift of 2 amino acids. Supporting the conservation of the 48 thus determined introns was the observation that they had a conserved phase in all cases except one. Experimentally-determined intron locations in *Paramecium *were kindly provided by Laurent Duret and Jean-François Goût (University de Lyon).

We compared the positions of the introns of *N. ovalis *against the position of the introns in their closest homologs (BlastX e-value threshold = 1.e-05) in another ciliate *T. thermophila *and a member of the Apicomplexa *P. falciparum*. Ciliates and Apicomplexa are both Alveolata. The genome of *T. thermophila *was recently published [3]. *Tetrahymena *protein and genomic sequences were obtained from the *Tetrahymena *Genome Database on . We worked with Assembly2 – Nov_2003_scaffolds and 27,769 cDNAs from 08-30-2006. By comparing the *N. ovalis *sequences to known proteins (Smith-Waterman against UniProt-Swissprot database; e-value threshold = 1e10-5), we identified homologs of the spliceosome complex.

### Motif, miRNA discovery

We used MEME [44], an expectation maximization algorithm and AlignACE [30], a Gibbs sampling program, to discover over-represented motifs de novo. We then tested their presence in the whole dataset using MAST [50].

We also used TRANSFAC [51], a database on eukaryotic *cis*-acting regulatory DNA elements and trans-acting factors that covers the whole range from yeast to human, to search these motifs in our set.

We detected tRNAs using tRNAscan-SE [43] with the default parameters. The potential presence of a miRNA was examined with the algorithm described in [73]

### Genetic Code

We determined the stop codon usage by examining those cDNAs that aligned with 1) at least 40% identity over 2) at least 60 amino acids that 3) did not have unknown nucleotides (N) in the alignment and 4) aligned until the N-terminus of a known protein. For the 328 cDNAs that fit those criteria we examined the next codon. TAA (141 cases) is preferentially used as stop codon, TAG (5 cases) also occurs in those locations, but less than e.g. GAC (40 cases) or GCC (59) cases.

### Phylogenetic analysis

rRNA sequences were extracted from Genbank; the sequence of the dinoflagellate *Symbiodinium *was used as an outgroup to root the tree.

Phylogenetic trees were constructed using an alignment provided by Muscle [74,75]. Sequences were edited and the most relevant parts from the alignments were selected manually using Seaview [76]. Phylogenies were subsequently derived using the program PhyML [77] using the HKY model and an estimated number of invariable sites with four substitution rate categories. 100 bootstraps were performed.

## Abbreviations

tRNA: transfer RiboNucleic Acid; TFBS: Transcription Factor Binding Site; nr: non-redundant; nt: nucleotide; SW: Smith-Waterman.

## Authors' contributions

GR did the main sequence analyses and drafted the manuscript. BD and ID determined the genetic code. RdG isolated and sequenced several complete genes and participated in drafting the manuscript. TvA, BB, GvdS, SM-vdS, isolated and sequenced several complete genes. AvH isolated and sequenced several complete genes and made southern blots. WJC sequenced 13 full length chromosomes, LFL supervised WJC and contributed to the manuscript as a whole and the genetic code discussion in particular. JH isolated the cells, initiated and coordinated parts of the study and participated in drafting the manuscript. MH supervised the sequence analysis, determined the intron conservation and finalized the manuscript.

## Supplementary Material

Additional file 1 ***N. ovalis *****introns found by comparison between cDNAs and gDNAs.** Comparison with the homologous sequences of ciliate *T. thermophila *and the apicomplexan *P. falciparum*. Click here for file

Additional file 2 **Selection of 3'UTRs.** Eight 3'UTRs carefully selected by hand. No polyadenylation signal can be observed. Click here for file
